# An Adsorptive Transfer Technique Coupled with Brdicka Reaction to Reveal the Importance of Metallothionein in Chemotherapy with Platinum Based Cytostatics

**DOI:** 10.3390/ijms11124826

**Published:** 2010-11-26

**Authors:** Sona Krizkova, Ivo Fabrik, Dalibor Huska, Vojtech Adam, Petr Babula, Jan Hrabeta, Tomas Eckschlager, Pavel Pochop, Denisa Darsova, Jiri Kukacka, Richard Prusa, Libuse Trnkova, Rene Kizek

**Affiliations:** 1 Department of Chemistry and Biochemistry, Faculty of Agronomy, Mendel University in Brno, Zemedelska 1, CZ-613 00 Brno, Czech Republic; 2 Department of Natural Drugs, Faculty of Pharmacy, University of Veterinary and Pharmaceutical Sciences, Palackeho 1-3, CZ-612 42 Brno, Czech Republic; 3 Department of Paediatric Haematology and Oncology, Charles University and Teaching Hospital Motol, 2nd Faculty of Medicine, V Uvalu 84, CZ-150 06 Prague 5, Czech Republic; 4 Department of Ophthalmology for Children and Adults, Charles University and Teaching Hospital Motol, 2nd Faculty of Medicine, V Uvalu 84, CZ-150 06 Prague 5, Czech Republic; 5 Department of Clinical Biochemistry and Pathobiochemistry, Charles University and Teaching Hospital Motol, 2nd Faculty of Medicine, V Uvalu 84, CZ-150 06 Prague 5, Czech Republic; 6 Department of Chemistry, Faculty of Science, Masaryk University, Kotlarska 2, CZ-611 37 Brno, Czech Republic

**Keywords:** tumor disease, metallothionein, platinum based anticancer drugs, anticancer therapy, resistance, retinoblastoma

## Abstract

The drugs based on platinum metals represent one of the oldest, but also one of the most effective groups of chemotherapeutic agents. Thanks to many clinical studies it is known that resistance of tumor cells to drugs is a frequent cause of chemotherapy failure. With regard to platinum based drugs, multidrug resistance can also be connected with increased expression of low-molecular weight protein metallothionein (MT). This study aimed at investigating the interactions of MT with cisplatin or carboplatin, using the adsorptive transfer technique coupled with differential pulse voltammetry Brdicka reaction (AdTS DPV Brdicka reaction), and a comparison of *in vitro* results with results obtained *in vivo*. The results obtained from the *in vitro* study show a strong affinity between platinum based drugs and MT. Further, we analyzed extracts of neuroblastoma cell lines treated with cisplatin or carboplatin. It is clear that neuroblastoma UKF-NB-4 cisplatin-resistant and cisplatin-sensitive cell lines unlikely respond to the presence of the platinum-based cytostatics cisplatin and carboplatin. Finally, we determined the level of MT in samples from rabbits treated with carboplatin and patients with retinoblastoma treated with the same drug.

## 1. Introduction

From a contemporary point of view, the process of the origin of malignant tumors is complex and multifactorial, comprising changes in expression of individual genes, physical factors and exposure to different chemical and biological impulses. Research and development in the field of tumor diagnostics and prevention, as well as methods of modern therapeutic approaches, belong to the priorities of all European Union countries. Individual tumor treatment strategies result from a number of treatment protocols combining an economic point of view and maximum benefit for the patient. One of the most important parts of the therapy is an efficacious chemotherapy application connected with the individualization of the therapy [1–10].

### 1.1. Cisplatin Based Chemotherapeutics

Drugs based on platinum metals represent one of the oldest, but also one of the most effective groups of chemotherapeutic agents [11–21]. Platinum-complex based chemotherapeutics are presently used in the therapy of a broad-spectrum of malignancies. The biological activity of the first-time-used platinum-based cytostatic agent, cisplatin, was discovered in 1965 by Rosenberg during research of the electric current influence on bacteria growth [22]. Since then, hundreds of platinum complexes have been synthesized and verified as anticancer chemotherapeutics, such as cisplatin, carboplatin, oxaliplatin, lobaplatin and ormaplatin, belonging to the best known. Though cisplatin has been successfully used in the therapy of malignant tumors for more than 25 years, its biochemical mechanism of action is still not clear. The cytotoxic effect of cisplatin consists in DNA adducts formation, especially to guanine and adenine, which causes DNA strands crossing and thereby inhibition of DNA synthesis and consequently mitosis. The therapeutic effect of platinum-based therapeutics is permanently irreplaceable and brings considerable benefits to patients [23–30].

### 1.2. Tumor Therapy Resistance

Thanks to many clinical research studies, it is known that resistance of tumor cells to drugs is a common cause of chemotherapy failure. This resistance develops via various mechanisms and the whole process is multifactorial at the cell level. Treatment of complications caused by multidrug resistance (MDR) is particularly difficult. One of the causes of tumor cell resistance is lowered therapeutic concentration of the cytostatic agent(s) in the target site. Membrane proteins such as Pgp, MRP and LRP participate in decreasing the cytostatics level in tumor cells. The complexity of MDR origination is also supported by changes in expression of genes participating in apoptosis or tumor suppressor genes. Cytostatics detoxification can be another factor leading to MDR, in which an increased activity of enzymes involved in oxidation or conjugation of xenobiotics with endogenic agents are most frequently found. In addition to these mechanisms, MDR can be also connected with the increased expression of low-molecular weight protein metallothionein (MT) [12,17–20,23,24,26,27,30–36].

### 1.3. Metallothionein Significance

**Figure 1.**
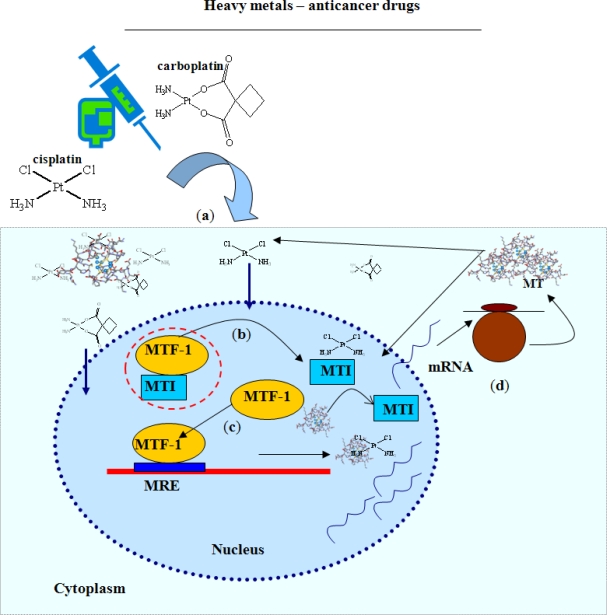
Simplified scheme of the interactions of MT with platinum-based cytostatics. (**a**) Application of platinum based cytostatic agents into organisms; (**b**) transport into the cell nucleus, (**c**) binding of platinum based cytostatic agents to metal transcription inhibitor (MTI) and release from complex with metal transcription factor-1 (MTF-1) followed by MTF-1 binding to metal responsive element (MRE). This step initiates mRNA MT synthesis. mRNA is consequently translated and active MT bind molecules of platinum-based cytostatics.

Metallothionein (MT) belongs to the group of intracellular, cysteine-rich proteins (Cys content up to 30% of the molecule) with a molecular weight of 6–10 kDa. The MT molecule consists of two binding domains composed of two cysteine clusters. The N-terminal part of the protein (β-domain) has three binding sites for divalent ions and the C-terminal protein part (α-domain) is able to bind four divalent metal ions. One MT molecule is able to bind 12 univalent and/or seven divalent metal ions. However, it is known that single MT molecules differ in their metal ions content and, thus, heavy metal binding sites in the MT molecule are not saturated under physiological conditions [37,38]. This is important for MT biological function (heavy metals homeostasis and detoxification, immunomodulation and regulation functions) [35,36,39,40]. One of the most important MT feature is its ability to interact with metal-containing therapeutics, such as platinum-based cytostatics. This ability can result in a decrease of the free cytostatic agent therapeutic level and thereby increase the resistance of tumor cells against cytostatic agent therapeutic concentrations. A review in Current Protein and Peptide Science aimed at clarifying the relationship between MT and tumor diseases was recently published [35]. Figure 1 shows a simplified scheme of interaction between MT and platinum-based cytostatics. Platinum-based therapeutic agents are probably able to bind to metal transcription inhibitor (MTI) that consequently releases the metal transcription factor-1 (MTF-1). Herewith, the MT biosynthesis is initiated and biosynthesized MT can interact with platinum-based cytostatic agent molecules. Thanks to inactivation of executive free therapeutic molecules by MT, the cytostatic agent therapeutic concentrations decreases and this leads to the growth of new cell subpopulations resistant to tumor therapy. The mechanisms of this process are still not clear [11,30,31,35,41–53]. This present study is aimed at studying interactions of metallothionein and cisplatin or carboplatin using the adsorptive transfer technique coupled with differential pulse voltammetry Brdicka reaction (AdTS DPV Brdicka reaction). We also aim to compare *in vitro* results with results obtained *in vivo*. Particularly, attention is paid to analysis of *in vitro* experiments, neuroblastoma cell line extracts, blood from rabbits treated with carboplatin, and patients with retinoblastoma treated with the same drug.

## 2. Results and Discussion

### 2.1. Significance of Thiol Compounds in Resistance Origination

It is known that thiol compounds play a very important role in the processes linked to the formation of tumor cell resistance to platinum-based cytostatics [35,54,55]. Recently, we suggested a unique electrochemical technique enabling direct MT detection in blood serum. This method is based on removal of interfering proteins by heat treatment and consequent adsorption of metallothionein to the surface of the working electrode [56,57].

### 2.2. Study of Platinum-Based Therapeutics Interactions with Metallothionein Modified Electrode

**Figure 2.**
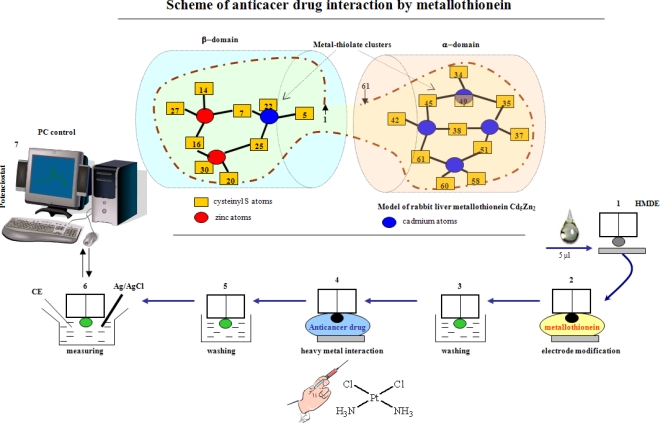
Scheme of study of the interaction between platinum-based cytostatics and MT. The MT molecule is composed of two domains containing cysteine clusters capable of binding metal ions. The hanging mercury drop electrode (HMDE) was immersed in a 5 μL drop of MT for 120 s (1 and 2), and after release of unbounded molecules (3), modified HMDE was immersed into a drop of cytostatic agent (4), then carefully rinsed with water. Electrochemical measurements followed (6) with computer processing of the data (7).

In the first *in vitro* experiment, we targeted the monitoring of cisplatin and carboplatin interactions with MT. We utilized an adsorptive transfer technique differential pulse voltammetry coupled Brdicka reaction (AdTS DPV Brdicka reaction) for this purpose. This technique has been successfully used for MT determination in numerous samples [57–60] and excellent correlation between immunochemical techniques was found [55,61]. The scheme of the experimental arrangement for the interaction studies is shown in Figure 2. A hanging mercury drop electrode (HMDE) was immersed in a 5 μL drop of MT for 120 s (point 1 and 2), after unbound molecules were released (3), the modified HMDE was immersed into a drop with a cytostatic agent (4). Then the electrode was carefully rinsed with water and analyzed (6) with computer treatment of the data obtained (7). Modification of the electrode and other experimental conditions were optimized in our previously published paper [62].

**Figure 3.**
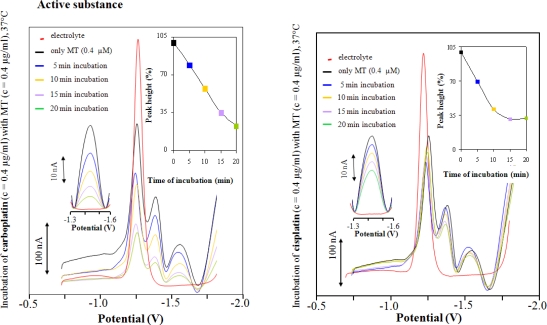
AdTS DP voltammograms of MT modified HMDE in the presence of the active cytostatic substances carboplatin (left panel) and cisplatin (right panel). MT and platinum-based cytostatics concentration 0.4 μg/mL; interaction time of MT with HMDE 120 s.

**Figure 4.**
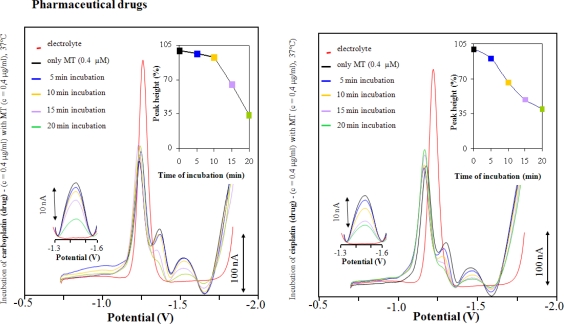
AdTS DP voltammograms of MT modified HMDE in the presence of pharmaceutical therapeutics containing cytostatic substances carboplatin (left panel) and cisplatin (right panel). MT and platinum-based cytostatics concentration 0.4 μg/mL; time of interaction MT with HMDE 120 s.

MT (0.4 μg/mL) modified HMDE was utilized for studying its interactions with cisplatin and carboplatin. For both compounds we obtained characteristic voltammograms (Figure 3). It clearly follows from the obtained results that the catalytic signal Cat2 diminished with increasing interaction time. After 20 min, the Cat2 signal decreased by more than 60% for both cytostatics. With increasing interaction times, no decrease of the signal was observed. For both drugs similar results were obtained, but the decrease of the signal was more rapid for cisplatin; where after 15 min interaction no further decrease of the signal was observed. For carboplatin, the decrease of the signal was slower and the lowering of the signal was complete after 20 min interaction. Similar results were obtained for interactions between cisplatin or carboplatin drugs, but a more distinct Cat2 signal decrease was observed after 10 min interaction for carboplatin and after 5 min for cisplatin (Figure 4).

The observed differences in the Cat2 signal decrease indicate that the studied compounds have different affinities for MT, probably due to their chemical structures. Differences between active substances and pharmaceutical drugs are probably caused by the presence of adjuvants in pharmaceutical drugs that significantly change their physicochemical properties (ionic strength, pH). Some of these parameters were investigated in our previous experiments [48], the obtained knowledge is possible to utilize for MT detection via hydrogen evolution. We can assume which platinum-based therapeutics (cisplatin, carboplatin) were captured by MT anchored onto HMDE surface and we were able to observe only changes directly connected with MT - drug interactions. In addition, El-Hourch *et al.* published the utilization of cisplatin for hydrogen evolution from supporting electrolyte [63]. However, platinum ions were not in the supporting electrolyte, thus they could not influence the catalytic reaction. The arrangement of the experiment also unambiguously points at the fact that carboplatin and cisplatin were strongly bound to the MT structure.

### 2.3. Changes in Cell Cultures

As it was demonstrated in our previous experiment *in vitro*, MT very rapidly and effectively binds platinum-based cytostatics (cisplatin and carboplatin). However, experiments carried out *in vitro* do not show the behavior of the analyzed compounds in an organism. Therefore, experiments *in vivo* were carried out to elucidate the significance of MT for resistance origination after the treatment by platinum-based therapeutics.

### 2.4. Automated Detection of Metallothionein

For routine analysis of larger sets of samples, an automated electroanalytical method for MT determination was found [48]. Reproducibility of determination varied within the range of 96 to 99% (n = 30). Dependence of studied MT signal height was linear in the concentration interval 0.1–5 μM. In our preliminary experiment, we demonstrated that the MT level increases in the presence of platinum-based cytostatics according to the applied concentration. Differences between cisplatin-resistant and cisplatin-sensitive tumor cell lines were also determined [48,55].

We were further interested in studying the time-dependence of these changes in greater detail. We compared the effect of cisplatin and carboplatin in concentrations of 0.01, 0.1 and 1 μM on resistant and sensitive tumor cell lines derived from neuroblastoma - UKF-NB-4. Cell lines were cultivated for 0.5, 1, 2, 4, 6, 12, 24 and 48 h in the presence of platinum-based cytostatic drugs such as cisplatin and carboplatin. At the end of the experiment, cells were homogenized and consequently MT level was determined in cell lysates using the procedure shown in [48].

**Figure 5.**
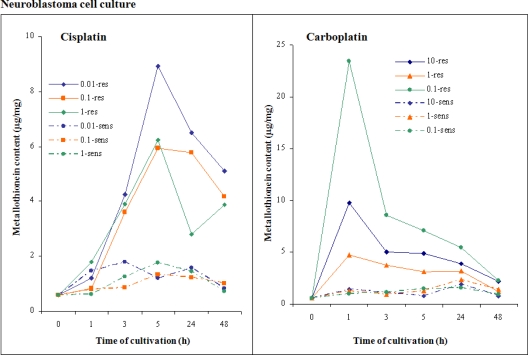
Neuroblastoma cell culture. Dependence of MT level in cell lines on the length of the cultivation. Prior to use in this experiment, all cell lines were cultivated for five sub-cultivations without cytostatics addition. Then, neuroblastoma cell cultures were exposed to cisplatin (left) or carboplatin (right).

Sensitive cell lines demonstrated a significant growth depression in the presence of cytostatic agents. MT level was significantly increased in all sensitive tumor cell lines by about 50–90% in comparison with the control cell line. As it is well evident from Figure 5, the MT level increased in resistant tumor cell lines compared to sensitive cells. In the resistant tumor cell lines, the increase of MT level was dependent on the cytostatic agent used and its concentration. With carboplatin, changes in MT level were observable from the beginning of the experiment, whereas under the two highest carboplatin concentrations the MT level was significantly increased in resistant tumor cells compared to sensitive ones. In the case of the cisplatin-resistant tumor cell line, the MT level increase was firstly observable after six hours of treatment. This increase was consequently followed by a moderate decrease in comparison with the resistant tumor cell line, whose maximum MT level was well evident already in the first hours of the experiment. Then, MT level relatively rapidly decreased to that of the values of sensitive tumor cell lines. Fluctuation in MT after cisplatin as well as carboplatin exposition with a similar development in the presence of all applied concentrations can be interpreted by initiatory free MT saturation by cytostatic agent and its resulting elimination from cells, which results in a decrease of MT level in cells. Resistant tumor cell lines, in contrast to the sensitive ones, responded to the cytostatic agent presence by significantly enhancing MT expression, which was well evident as a rapid increase of the MT level. Determined differences in MT levels during platinum-based cytostatics treatment are in good agreement with our knowledge about cytostatics detoxification via thiol compounds that increase the ability of these tumor cell lines to survive in the presence of platinum-based therapeutics. From the obtained results, it clearly follows that neuroblastoma UKF-NB-4 cisplatin-resistant and cisplatin-sensitive cell lines unlikely respond to the presence of platinum-based cytostatics cisplatin and carboplatin.

### 2.5. Changes of MT Level in Rabbits after Intravitreal Carboplatin Administration

**Figure 6.**
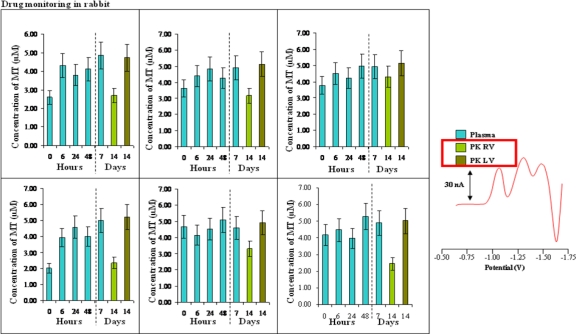
Time-dependent changes in concentrations of MT in the plasma of rabbits treated with carboplatin. The increase of MT levels is observed after injection of drug. PK RV (peripheral blood from right ventricle) has always higher MT concentration than PK LV (peripheral blood from right ventricle). Typical DP voltammogram of plasma analysis is shown on the right side.

Retinoblastoma is one of the most frequent intraocular malignant tumors and it is the malignancy with the highest incidence in children. Retinoblastoma genesis is connected with gene mutation of both alleles of the retinoblastoma gene RB1 localized on chromosome 13q1.4. Its therapy is still very difficult, thus, new therapeutic possibilities as well as new ways of cytostatics application are intensively searched. One of these applications is direct injection of cytostatics—in this case especially carboplatin—into the eye. For this purpose, carboplatin was applied to experimental animals and consequently MT level was determined using automated electrochemical detection. Figure 6 shows the changes in MT levels according to the length of the carboplatin treatment. At the beginning, the MT level was about 3.5 μM in healthy rabbits. After carboplatin application, a significant increase in the MT level was determined in all experimental animals up to 5 μM. An enhanced MT level was detected for the following seven days in all experimental animals. A slight decrease in MT level was observed 14 days after carboplatin application. From the obtained results, it clearly follows that MT level was induced by carboplatin application directly into the eyes and can be probably connected with the resistance origination.

### 2.6. MT Changes in Retinoblastoma Patients

**Figure 7.**
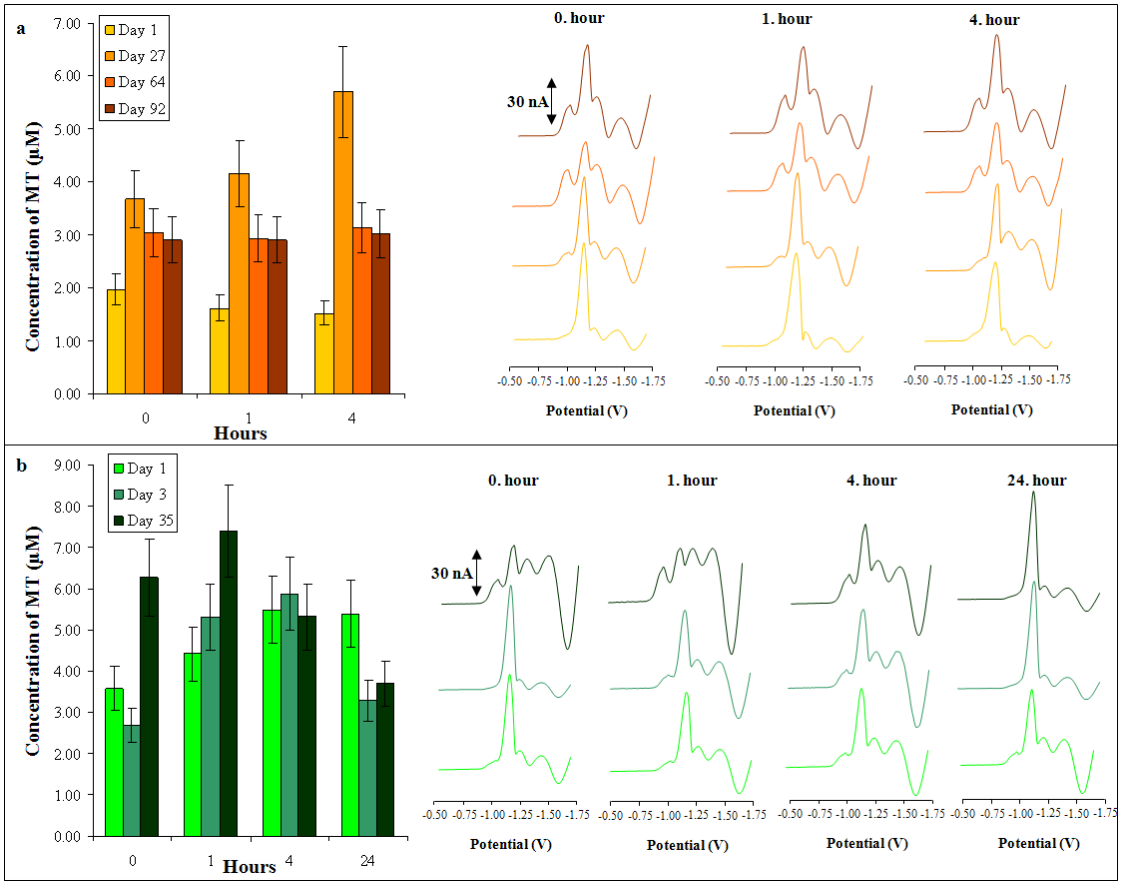
Time-dependent changes in the concentration of MT in the plasma of two patients (**a**, **b**) with retinoblastoma. MT levels were monitored 4 or 24 hours after application of the drug and the same procedure was repeated after a few days during the next treatment with carboplatin. DP voltammograms are shown on the right.

Retinoblastoma is a very rare malignant disease in the Czech Republic. Its treatment is centralized into a specialized workplace: FN Motol, Prague. Samples of retinoblastoma patients (n = 2) treated long-term with carboplatin were analyzed. Figure 7 shows the changes in MT level during long-term therapy in relation to the time-dependent changes after cytostatic agent application. MT levels were monitored 4 or 24 hours after application of the drug and the same procedure was repeated after a few days during the next treatment with carboplatin. In short-time monitoring of MT concentrations, a higher increase was observed with each dose of carboplatin; nevertheless, long periods had a stabilization effect on MT levels in the plasma after carboplatin application.

Immediately after carboplatin application, no MT level increase was detected; only in the first minutes after application a moderate MT level decrease was determined. This is probably connected with the interaction with carboplatin and free MT. Three days after the first application of carboplatin, a moderate MT level decrease (about 9%) was observed in comparison with the first application. Application after 27 days since the first application led to a significant MT level enhancement (165%) compared to the first day. Maximal MT levels were determined between the 30^th^ and 60^th^ day after initial carboplatin application. After the 60^th^ day after the first carboplatin application, MT levels slowly decreased. Figure 7 shows characteristic voltammetric records of individual samples with well distinguishable signals.

## 3. Experimental Section

### 3.1. Chemicals and pH Measurements

Rabbit liver MT (MW 7143), containing 5.9% Cd and 0.5% Zn, was purchased from Sigma Aldrich (St. Louis, MO, USA). Tris(2-carboxyethyl)phosphine (TCEP) was produced by Molecular Probes (Evgen, Oregon, WI, USA). Other chemicals used were purchased from Sigma Aldrich. The stock standard solutions of MT at 10 μg/mL with 1 mM TCEP were prepared with ACS water (Sigma-Aldrich, St. Louis, MO, USA) and stored in the dark at −20 °C. Working standard solutions were prepared daily by dilution of the stock solutions. The pH was measured using pH meter WTW inoLab (Weilheim, Germany). The pH-electrode (SenTix-H, pH 0–14/3M KCl) was regularly calibrated by a set of WTW buffers (Weilheim, Germany).

### 3.2. Tumor Cell Lines

The following cell lines obtained from the Department of Paediatric Haematology and Oncology, Charles University, Prague, Czech Republic were used: UKF-NB4—cisplatin or carboplatin sensitive (prepared from recurrence of neuroblastoma into bone marrow, with MYCN amplification, del 1p34.2ter, del 13iso 17q) and UKF-NB4—cisplatin or carboplatin resistant (the line derived from the previous line—UKF-NB4 with *in vitro* induced resistance to cisplatin or carboplatin). The cell lines were prepared by cultivation with increasing concentration of cisplatin or carboplatin. The cells were cultivated in IMDM medium with 10% fetal calf serum at 37 °C, the chemoresistant cell lines were cultivated in medium with cisplatin or carboplatin added.

### 3.3. Human Blood Serum

The samples of blood were obtained using vein tapping to the closed tapping units without another reagent during medical treatment of two patients with malignancies at Teaching Hospital Motol, Czech Republic. All patients subscribed to informed consent with utilization of their blood samples for the research.

### 3.4. Preparation of the Samples for Electroanalytical Determination of Metallothionein

#### 3.4.1. Human Blood Serum

Human blood serum samples were prepared by heat treatment and solvent precipitation. Briefly, samples were kept at 99 °C in a thermomixer (Eppendorf 5430, USA) for 15 min with occasional stirring, and then cooled to 4 °C. The denatured homogenates were centrifuged at 4 °C, 15,000 × g for 30 min (Eppendorf 5402, USA). Heat treatment effectively denatures and removes high molecular weight proteins from samples [56]. Determination of MT in the human blood serum samples was performed by differential pulse voltammetry Brdicka reaction. Analyzed sample volume was 5 μL.

#### 3.4.2. Tumor Cell Lines

The harvested cells were transferred to a test tube and deep frozen in liquid nitrogen to disrupt the cells. The frozen cells were mixed with extraction buffer (100 mM potassium phosphate, pH 8.7) to a final volume of 1 mL and homogenized using hand-operated homogenizer ULTRA-TURRAX T8 (IKA, Germany) placed in an ice bath for 3 min at 25,000 rpm. The homogenate was centrifuged at 10,000 × g for 15 min and at 4 °C (Eppendorf 5402, USA) [64]. The supernatant were processed in the same way as the human blood serum samples mentioned in Section 3.4.1. The processed samples were measured by adsorptive transfer stripping technique coupled with chronopotentiometric stripping analysis.

#### 3.4.3. Intravitreal Administration of Carboplatin in Rabbits

Administration of carboplatin was performed according to data which indicate that periocular or intravitreal (transcorneal) carboplatin injections rather than systematically administrated carboplatin can effectively treat children with intraocular retinoblastoma. Six adult New Zealand White Rabbits (Anlab, Czech Republic) with an average weight of 2.45 kg (SD ± 0.38) were handled according to the statement Nr.207/2004 s.11 of the Ministry of Agriculture of the Czech Republic for the use of animals in experiments. *In vivo* experiments were further approved by the ethical committee for animal welfare of Charles University, 2nd Faculty of Medicine. The animals were kept at 22–25 °C room temperature, at 55–65% relative air humidity, and on a 12 h light/dark cycle, and were provided with tap water and rabbit chow TM MaK 1 (Bergman, Kocanda, Czech Republic) *ad libitum*. The rabbits were anesthetized intramuscularly with a mixture of 30–50 mg/kg of ketamine hydrochloride (Narketan 10, Vétoquinol, France) and 5 mg/kg of xylazine hydrochlorid (Rometar 2%., Spofa, Czech Republic) throughout the experiment. Topical oxybuprocaine eye drops (0.4%, Benoxi, Unimed Pharma, Slovakia) were used to anesthetize the ocular surface before intravitreal puncture.

Carboplatin (Carboplatin, TEVA, Netherlands) solution was prepared immediately before injection by dissolving carboplatin in sterile water with mannitol to the final used concentrations. A single transcorneal intravitreal injection of carboplatin (0.05 mg in total volume of 0.1 mL) was applied with a 25-gauge needle into the right eye; the left eye of all animals served as uninjected controls. Blood samples were taken at 0, 6, 24 and 48 h, 7 and 14 days after injection. Blood samples were drawn from the peripheral auricular vein and collected in tubes with ammonium heparinate. The blood was centrifuged at 3,000 rpm for 10 minutes to separate blood plasma.

### 3.5. Electrochemical Measurements

#### 3.5.1. Electroanalytical Determination of Metallothionein

An adsorptive transfer stripping technique (AdTS) coupled with DPV Brdicka reaction was employed for the determination of metallothionein in the cell lines extracts. The electrochemical measurements were performed using an AUTOLAB analyzer (EcoChemie, The Netherlands) connected to VA-Stand 663 (Metrohm, Switzerland), using a standard electrochemical cell with a classical three-electrode connection. The three-electrode system consisted of a hanging mercury drop electrode as the working electrode, an Ag/AgCl/3 M KCl reference electrode and a glassy carbon auxiliary electrode. For smoothing and baseline correction, the software GPES 4.9 supplied by EcoChemie was employed. The Brdicka supporting electrolyte (1 mM Co(NH_3_)_6_Cl_3_ in 1 M ammonia buffer (NH_3_(aq) + NH_4_Cl, pH = 9.6) was used; no surface-active agent was added. AdTS DPV Brdicka reaction parameters were as follows: initial potential of −0.6 V, end potential −1.6 V, modulation time 0.057 s, time interval 0.2 s, step potential of 1.05 mV, modulation amplitude of 250 mV, E_ads_ = 0 V. Temperature of the supporting electrolyte was 4 °C.

#### 3.5.2. Automated Electroanalytical Determination of Metallothionein

Differential pulse voltammetric Brdicka’s reaction measurements were performed with 747 VA Stand instrument connected to 746 VA Trace Analyzer and 695 Autosampler (Metrohm, Switzerland), using a standard cell with three electrodes and a cooled sample holder (4 °C). A hanging mercury drop electrode (HMDE) with a drop area of 0.4 mm^2^ was the working electrode. An Ag/AgCl/3M KCl electrode was the reference and glassy carbon electrode was the auxiliary electrode. The software GPES 4.9 supplied by EcoChemie was employed for smoothing and baseline correction of the raw data. The analyzed samples were deoxygenated prior to measurements by purging with argon (99.999%), saturated with water for 120 s. The Brdicka’s supporting electrolyte containing 1 mM Co(NH_3_)_6_Cl_3_ and 1 M ammonia buffer (NH_3_(aq) + NH_4_Cl, pH = 9.6) was used and after each measurement the supporting electrolyte was exchanged. The parameters of the measurement were as follows: initial potential of −0.7 V, end potential of −1.75 V, modulation time 0.057 s, time interval 0.2 s, step potential 2 mV, modulation amplitude −250 mV, E_ads_ = 0 V. All experiments were carried out at 4 °C employing thermostat Julabo F12 (Labortechnik GmbH, Germany). One hundred-times diluted sample with 0.1 M phosphate buffer pH 7.0 (20 μL) was used.

### 3.6. Descriptive Statistics

Data were processed using MICROSOFT EXCEL® (USA) and STATISTICA.CZ Version 8.0 (Czech Republic). Results are expressed as mean ± standard deviation (S.D.) unless otherwise noted (EXCEL^®^). Statistical significance of the differences between MT levels and rat weight were determined using STATISTICA.CZ. Differences with p < 0.05 were considered significant and were determined using one way ANOVA test (particularly Scheffe test), which was applied for means comparison.

## 4. Conclusions

Adsorptive transfer technique differential pulse voltammetry coupled with Brdicka reaction presents a unique possibility for studying interactions between proteins and cytostatics. Catalytic signals are very sensitive and enable the study of interactions at the nanomolar level. In addition, our *in vivo* experiments verify the hypothesis that MT levels significantly increase after platinum-based therapeutic application. In the case of platinum-resistant tumor cell lines, the MT level significantly increases. Thus, MT can act as one of the factors of tumor therapy resistance. For understanding these mechanisms, experiments on experimental animals were carried out and in addition MT levels were monitored in patients with a rare malignant disease—retinoblastoma—after platinum-based cytostatics treatment.
